# The Sterolgene v0 cDNA microarray: a systemic approach to studies of cholesterol homeostasis and drug metabolism

**DOI:** 10.1186/1471-2164-9-76

**Published:** 2008-02-11

**Authors:** Tadeja Režen, Peter Juvan, Klementina Fon Tacer, Drago Kuzman, Adrian Roth, Denis Pompon, Lawrence P Aggerbeck, Urs A Meyer, Damjana Rozman

**Affiliations:** 1 Center for Functional Genomics and Bio-Chips, Institute of Biochemistry, Faculty of Medicine, University of Ljubljana, Zaloška 4, SI-1000 Ljubljana, Slovenia; 2 Artificial Intelligence Laboratory, Faculty of Computer and Information Science, University of Ljubljana, Tržaška cesta 25, SI-1000, Slovenia; 3 Lek Pharmaceuticals d.d., Verovškova 57, SI-1000 Ljubljana, Slovenia; 4 Genome Scale Biology, Biozentrum, University of Basel, Klingelbergstrasse 70, CH-4056 Basel, Switzerland; 5 CNRS, Avenue de la Terrasse, F-91198 Gif-sur-Yvette, France

## Abstract

**Background:**

Cholesterol homeostasis and xenobiotic metabolism are complex biological processes, which are difficult to study with traditional methods. Deciphering complex regulation and response of these two processes to different factors is crucial also for understanding of disease development. Systems biology tools as are microarrays can importantly contribute to this knowledge and can also discover novel interactions between the two processes.

**Results:**

We have developed a low density Sterolgene v0 cDNA microarray dedicated to studies of cholesterol homeostasis and drug metabolism in the mouse. To illustrate its performance, we have analyzed mouse liver samples from studies focused on regulation of cholesterol homeostasis and drug metabolism by diet, drugs and inflammation. We observed down-regulation of cholesterol biosynthesis during fasting and high-cholesterol diet and subsequent up-regulation by inflammation. Drug metabolism was down-regulated by fasting and inflammation, but up-regulated by phenobarbital treatment and high-cholesterol diet. Additionally, the performance of the Sterolgene v0 was compared to the two commercial high density microarray platforms: the Agilent cDNA (G4104A) and the Affymetrix MOE430A GeneChip. We hybridized identical RNA samples to the commercial microarrays and showed that the performance of Sterolgene is comparable to commercial arrays in terms of detection of changes in cholesterol homeostasis and drug metabolism.

**Conclusion:**

Using the Sterolgene v0 microarray we were able to detect important changes in cholesterol homeostasis and drug metabolism caused by diet, drugs and inflammation. Together with its next generations the Sterolgene microarrays represent original and dedicated tools enabling focused and cost effective studies of cholesterol homeostasis and drug metabolism. These microarrays have the potential of being further developed into screening or diagnostic tools.

## Background

The level of cholesterol in mammals is tightly regulated by multiple homeostatic mechanisms which affect cholesterol biosynthesis, metabolism and uptake-excretion cycles. This regulation is achieved by different transcriptional factors, including sterol regulatory element-binding proteins (SREBP) and different members of the nuclear receptor superfamily [1,2]. Under certain conditions (inflammation, high-cholesterol diet, etc.) cholesterol homeostasis is disrupted and can lead to atherosclerosis and other cardiovascular diseases, gallstone formation or neurological disorders. Cardiovascular diseases remain a leading cause of death in the developed world and are therefore an important research area. The central question is how different physiological processes being provoked by endogenous and exogenous sources influence cholesterol homeostasis and what the key targets of these regulations are. These findings are also of crucial importance for the development of novel hypolipidemic drugs.

Each exogenous compound entering the body requires detoxification by drug metabolism enzymes from different families. This process is also regulated by members of the nuclear receptor superfamily [3]. Recent studies have shown important crosstalk between drug metabolism and cholesterol homeostasis through nuclear receptor-mediated pathways [4]. However, this complex regulatory network is still far from being completely understood.

Microarray technology is a powerful tool and has gained broad usage in basic and medical research. It enables systemic studies of gene expression of selected genes or of the entire genome and can lead to the discovery of new genes involved in biological processes. Several commercial and academic DNA microarrays are currently available. Commercially available are often whole-genome arrays, which are not cost-effective for studies of a small number of selected genes [5]. This provides a rationale for developing small-scale arrays, enabling cost-effective studies of a limited number of genes involved in specific biochemical pathways [6-9], thus allowing more detailed studies of specific biological processes. Commercial arrays often do not include genes which may be of a particular interest to specific users [10-12]; neither do they always offer control over probe sequences. In the development process of a small-scale custom array special care is taken in the selection of the studied genes and specifically the position of their probes. The rationale for designing custom arrays is in the ease of their adaptation, e.g. addition of new genes/probes, removal of non-working probes and modification of the geometry. Owing to low production costs and small overhead with annotations the adaptations can be performed on the fly during experimentation, which is unfortunately still not the case with commercially available arrays.

We have developed Sterolgene v0 cDNA microarray as a prototype in a series of Sterolgene/Steroltalk cDNA microarrays. These dedicated arrays enable focused studies of cholesterol homeostasis and drug metabolism in mouse and in human. We have validated the Sterolgene array in studies of experimental disturbances of cholesterol homeostasis by inflammation, diet and drugs in the mouse liver. We compared the results to two commercial platforms: the Agilent cDNA (G4104A) and the Affymetrix MOE430A GeneChip. Within this paper we show that the Sterolgene array performs as well as any of the two commercial platforms in terms of detection of changes in expression of genes of our interest from the cholesterol homeostasis and drug metabolism induced by the studied factors.

## Results

### Production of the Sterolgene v0 microarray

**Figure 1:**
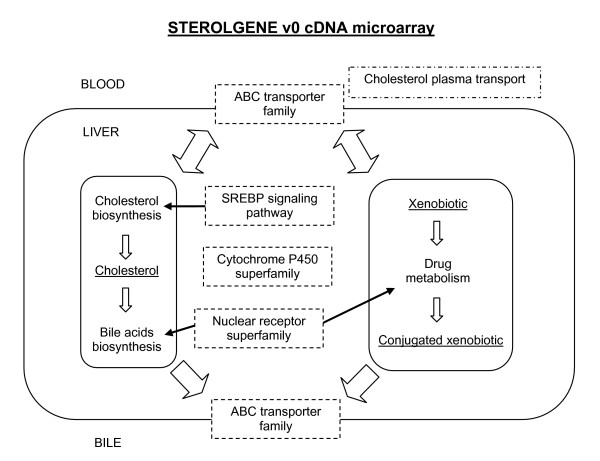
**Drug metabolism and cholesterol homeostasis**. Main groups of genes involved in cholesterol homeostasis and drug metabolism present on the Sterolgene v0 cDNA microarray. A scheme of the liver cell is shown together with its surroundings (blood, bile).

**Figure 2:**
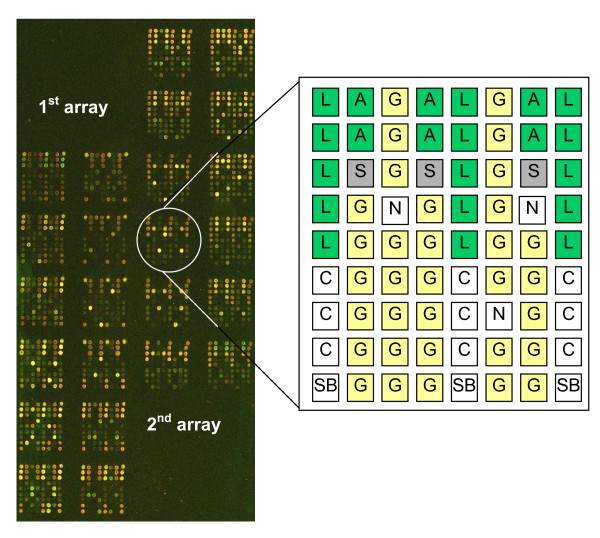
**Sterolgene v0 microarray design**. Complete array containing 12 blocks was spotted twice to increase number of replicas. Each block contains positive and negative controls to enable spatial normalization. Each gene probe is spotted three times in two blocks. Green spots – positive controls: L – Firefly luciferase, A – ArrayControls Spikes (Ambion); white spots – negative controls: C – bacterial gene probe, SB – spotting buffer, N – empty spot; grey spots – salmon sperm DNA (S); yellow spots – gene probes (G).

The Sterolgene array contains 95 mouse genes known to be involved in cholesterol homeostasis and drug metabolism. Among these are members of the cytochrome P450 superfamily, the nuclear receptor superfamily, the ABC transporters and other transporter families, genes for cholesterol biosynthesis and SREBP signaling and some other genes linked to cholesterol homeostasis (Figure 1). The complete gene list with corresponding accession numbers is available in Additional file 1. The positions of probes have been selected according to *a priori *knowledge of each gene. Probes detect all gene transcripts known at the time of design. In case of major differences between transcripts more probes per gene were selected. Each gene probe is spotted in 6 replicas, three in two blocks. Each block contains 23 types of positive controls (most of them spotted in 6 replicas) and 3 types of negative controls (Figure 2). These serve as control of quality; positive controls are also used for data normalization. Level of unspecific cross-hybridization, background intensity and uniformity were used to optimize hybridization and washing protocols.

### Normalization of Sterolgene data

**Figure 3:**
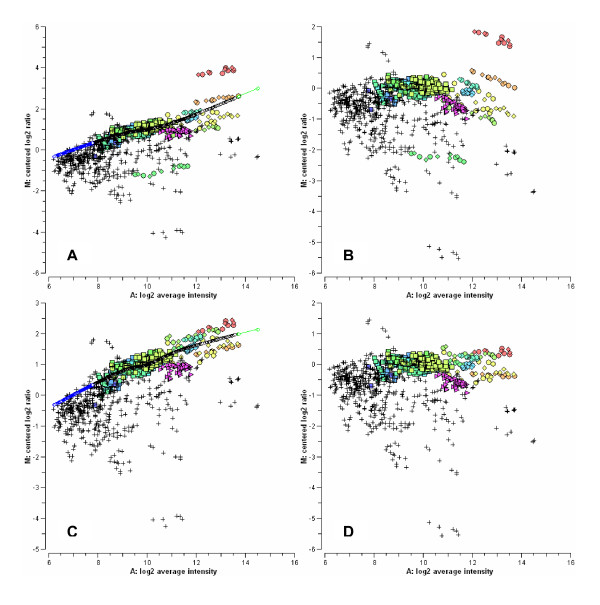
**Normalization of Sterolgene v0 microarray data**. MA plots of Sterolgene v0 microarray data illustrating its normalization. Genes are marked with crosses (+), Ambion ArrayControl spikes with circles (○) and diamonds (◇), and Firefly luciferase mRNAs with squares (□). A: data before normalization together with LOWESS curve through normalization spike in RNAs. C: the same data as shown in A, but with normalization controls adjusted according to their RNA concentrations (see text for details). B and D: data after normalization according to LOWESS curves shown in A and C, respectively. Adjusted are 4 types of Ambion spikes marked with green, orange and red circles/diamonds.

Normalization of Sterolgene data was done using LOWESS fit to normalization spike in RNAs according to their average intensity (*A *= *log*_2_√(R*G), where *R *and *G *represent signal intensities). Figure 3A shows a MA plot of Sterolgene data and a LOWESS curve fitted to the normalization controls. However, data from normalization controls were first adjusted according to concentrations in which normalization RNAs are added to sample RNAs: log_2 _ratios of concentrations are subtracted from the log_2 _ratios of measured intensities (*M *= *log*_2_(*R*/*G*)). In Figure 3C the same data as in 3A is shown but with normalization controls adjusted according to their RNA concentrations. Notice the difference between the normalization curves at large *A *values. Such adjustment results in better fit of the normalization curve and consequently in more accurate normalization. Figures 3B and 3D show MA plots of data normalized according to the LOWESS curves shown in Figures 3A and 3C, respectively. Notice the difference in *M *for genes with large *A *values. Sterolgene data was normalized in Orange software [13] using a dedicated normalization widget, which was developed specifically for normalization of custom low-density arrays.

### Acute TNFα inflammation and fasting affect cholesterol homeostasis and drug metabolism

The Sterolgene microarray was first evaluated in studies of how acute inflammation affects cholesterol homeostasis and drug metabolism. Mouse liver RNAs from the fasting and TNF-α/fasted experiments were hybridized to Agilent 10 K cDNA microarrays (G4104A) and Sterolgene arrays according to the recommended protocols. Differential expression was determined by two tailed Student's t-test. For Sterolgene data, probability of type I error α_S _= 0.05 was used. For Agilent data, a complementary probability of type I error was calculated according to Bonferroni correction for multiple testing (α_A _= α_S_*n_S_/n_A _= 0.00055 where n_S _and n_A _represent the number of genes included in Sterolgene and Agilent arrays, respectively), but final analysis was made using a more relaxed criteria α_A _= 0.01.

**Figure 4:**
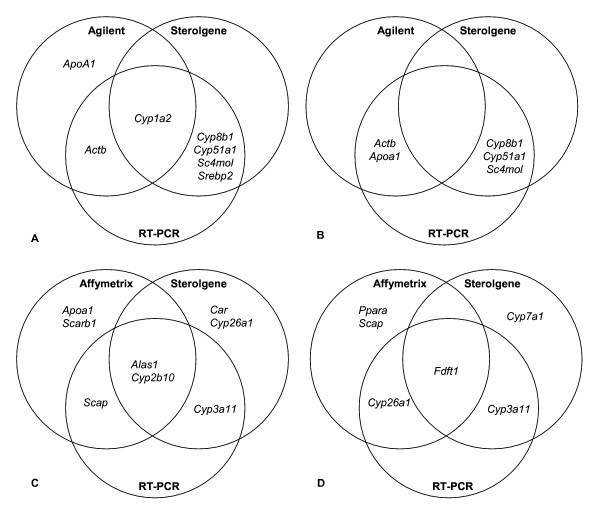
**Agreement of Sterolgene, Agilent and Affymetrix with RT-PCR data**. Venn diagrams illustrating genes differentially expressed by Sterolgene, Agilent, Affymetrix and RT-PCR measurements. Only genes tested by RT-PCR are shown. A: fasting experiment, B: TNF-α experiment, C: phenobarbital experiment, D: cholesterol feeding experiment. Differential expression was determined using two tailed Student's t-test at probability of type I error α = 0.05 for Sterolgene-Agilent comparison (A and B) and α = 0.1 for Sterolgene-Affymetrix comparison (C and D). Differential expression in RT-PCR experiments was determined using two tailed Student's t-test at probability of type error α = 0.05.

Using the Sterolgene array we show that fasting turns off most of anabolic processes due to energy conserving shifts in the liver and we observe a down-regulation of a majority of genes. These genes code for drug metabolizing enzymes (*Cyp3a41*, *Cyp1a2*, *and Cyp3a25*), different nuclear receptors (*Pparα*, *Rorγ*, *Rorα*, *and Lxrα*), cholesterol biosynthesis (*Acat2*, *Mvk*, *Sc4mol*, *Cyp51a1*, *Nsdhl*, *Sc5d*,) and transcriptional regulation of cholesterol biosynthesis (*Srebp2*) (Table 1). We also observed down-regulation of *Alas1 *(delta-aminolevulinate synthase 1) and *Cyp11a1*, which has previously been reported [14]. However, bile acid synthesis (*Cyp8b1*) is up-regulated by fasting. Three down-regulated (*Cyp51a1*, *Sc4mol*, *Srebp2*) and one up-regulated (*Cyp8b1*) gene were confirmed by RT-PCR [15] (Figure 4A).

**Table 1:** Differentially expressed genes in the mouse liver after fasting (Sterolgene v0 microarray). Differentially expressed genes in the mouse liver after fasting as detected by the Sterolgene array (α = 0.05).

**Gene name and function**	**Gene symbol**	**Log_2 _ratio**	**GeneBank Acc. No.**
*Cholesterol biosynthesis*			
Acetyl-CoA acetyltransferase 2	Acat2	-1.13	NM_009338
Mevalonate kinase	Mvk	-0.56	NM_023556
**Lanosterol 14α-demethylase**	**Cyp51a1**	**-1.32**	BC031813
**Sterol-C4-methyl oxidase-like**	**Sc4mol**	**-2.05**	NM_025436
NAD(P) dependent steroid	Nsdhl	-1.47	BC019945
dehydrogenase-like			
Sterol C5 desaturase	Sc5d	-1.75	BC024132
*Bile acid biosynthesis*			
**Sterol 12α-hydroxylase**	**Cyp8b1**	**0.49**	NM_010012
*Cytochrome P450 superfamily*			
Cytochrome P450, 1a2	Cyp1a2	-1.35	NM_009993
Cytochrome P450, 3a25	Cyp3a25	-0.78	BC028855
Cytochrome P450, 3a41	Cyp3a41	-1.91	NM_017396
Cholesterol side chain cleavage enzyme	Cyp11a1	0.41	NM_019779
Retionic acid hydrolase	Cyp26a1	-0.83	NM_007811
*Nuclear receptor superfamily*			
Peroxisome proliferator-activated	Nr1c1	-1.03	NM_011144
receptor alpha			
Retinoic acid-related orphan	Nr1f3	-0.60	NM_011281
receptor gamma			
Retinoic acid-related orphan	Nr1f1	-0.54	NM_013646
receptor alpha			
Liver X receptor beta	Nr1h2	-0.46	NM_009473
Constitutive androstane receptor	Nr1i3	0.22	NM_009803
*Transcription factors*			
**Sterol regulatory element binding transcription factor 2**	**Srebp2**	**-0.60**	NM_033218
CCAAT/enhancer binding protein alpha	Cebpa	-0.38	BC028890
Transcription factor 1	Tcf1	0.17	NM_009327
*Other*			
Peptidylprolyl isomerase A	Ppia	-0.65	NM_008907
Organic anion transporter 1	Slco1a1	-0.55	AY195868
Delta-aminolevulinate synthase 1	Alas1	0.98	NM_020559

Genes in bold – RT-PCR analysis is consistent with microarray data. Cyp3a41 probe cross-hybridizes with Cyp3a11.

Note. Three mice were used in this experiment.

In TNF-α treated fasted mice we observe up-regulation of cholesterol biosynthesis (*Cyp51a1*, *Sc4mol*) and down-regulation of cholesterol excretion (*Cyp8b1*) (Table 2). However, drug metabolism is further repressed (*Cyp2f2*, *Cyp2j5*, *Cyp3a25*) and this also has previously been shown [16]. Two up-regulated (*Cyp51a1*, *Sc4mol*) and one down-regulated gene (*Cyp8b1*) from cholesterol homeostasis have been confirmed by RT-PCR [15] (Figure 4B).

**Table 2:** Differentially expressed genes in the mouse liver after TNF-α treatment and fasting (Sterolgene v0 microarray). Differentially expressed genes in the mouse liver after TNF-α treatment/fasting as detected by the Sterolgene array (α = 0.05). Genes in bold – RT-PCR analysis is consistent with microarray data.

**Gene name and function**	**Gene symbol**	**Log_2 _ratio**	**GeneBank Acc. No.**
*Cholesterol biosynthesis*			
**Lanosterol 14α-demethylase**	**Cyp51a1**	**1.35**	BC031813
**Sterol-C4-methyl oxidase-like**	**Sc4mol**	**1.63**	NM_025436
*Bile acid biosynthesis*			
**Sterol 12α-hydroxylase**	**Cyp8b1**	**-1.82**	NM_010012
*Cytochrome P450 superfamily*			
Cytochrome P450, 2f2	Cyp2f2	-1.72	NM_007817
Cytochrome P450, 2j5	Cyp2j5	-0.68	NM_010007
Cytochrome P450, 3a25	Cyp3a25	-1.56	BC028855
Cholesterol side chain cleavage enzyme	Cyp11a1	-0.30	NM_019779
*Nuclear receptor superfamily*			
Testis receptor	Nr2c1	-1.31	NM_011629
Glucocorticoid receptor	Nr3c1	-0.97	NM_008173
Androgen receptor	Nr3c4	-0.71	M37890
*Other*			
Glyceraldehyde-3-phosphate dehydrogenase	Gapd	0.53	NM_008084

Note. Three mice were used in this experiment.

With the Agilent array we were not able to detect any change in cholesterol homeostasis and drug metabolism caused by TNF-α and fasting (Additional files 2 and 3). Agreement between both platforms is low with only one gene in common in TNF-α/fasted experiment (*Cyp2f2*) and with no genes in common in fasting experiment.

Additionally, we have selected only genes that are present in both platforms (we found 54 such genes) and determined their differential expression using the same p-value cut-off as for Sterolgene data (α = 0.05). The rationale behind was not to tighten the analysis conditions for Agilent data just due to the fact that the Agilent array contains more genes than the Sterolgene array. In fasting experiment we found 5 genes differentially expressed (Additional file 4) and of these *Cyp1a2 *was confirmed by Sterolgene and RT-PCR, *Actb *by RT-PCR, while *Apoa1 *was not confirmed by RT-PCR [15] (Figure 4A). In TNF-α/fasted experiment we found 4 genes differentially expressed (Additional file 5) and of these *Cyp2f2 *was confirmed by the Sterolgene (RT-PCR not performed), and *Actb *and *Apoa1 *by RT-PCR [15].

**Figure 5:**
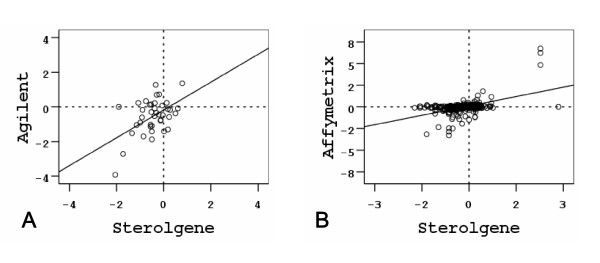
**Scatterplots of log_2 _ratios between Sterolgene, Agilent and Affymetrix**. Scatterplots of log_2 _ratios of genes that are present in Agilent and Sterolgene arrays (A), and Affymetrix and Sterolgene arrays (B). Values from both experiments performed for each cross-platform comparison were combined in a single plot.

Low correspondence in differential expression may be due to the fact that of 54 genes in common only 20 showed significant signal on the Agilent arrays. For these genes we calculated Pearson's product moment correlation coefficient between log_2 _ratios in both experiments. Figure 5A shows a scatterplot of that data. Though the correspondence in differential expression is low, we observed a significant correlation between the platforms (r = 0.508, p = 0.001, N = 40), which is comparable to correlations reported by other cross-platform comparison studies [12,17-19].

### Phenobarbital and cholesterol feeding affect cholesterol homeostasis and drug metabolism

The Sterolgene array was also tested in studies of how phenobarbital and cholesterol feeding affect cholesterol homeostasis and drug metabolism. Mouse liver RNAs were hybridized to Affymetrix MOE430A GeneChips and Sterolgene arrays according to the recommended protocols. Differential expression was determined by two tailed Student's t-test. For Sterolgene data, probability of type I error α_S _= 0.1 was used. For Affymetrix data, a complementary probability of type I error was calculated according to Bonferroni correction for multiple testing (α_Af _= α_S_*n_S_/n_Af _= 0.00043 where n_S _and n_Af _represent the number of genes included in Sterolgene and Affymetrix arrays, respectively), but final analysis was made using a more relaxed criteria α_A _= 0.001.

In phenobarbital experiment, we detected several previously known up-regulated genes (*Cyp2b *and *Cyp3a *families, *Cyp2a4 *and *Alas1*) [20-22] using the Sterolgene array (Table 3). However, among these only *Cyp2b10 *was detected using Affymetrix arrays (Additional file 6). *Alas1*, *Cyp2b10 *and *Cyp3a11 *were confirmed by RT-PCR (Figure 4C).

**Table 3:** Differentially expressed genes in the mouse liver after phenobarbital treatment (Sterolgene v0 microarray). Differentially expressed genes in the mouse liver after phenobarbital treatment as detected by the Sterolgene array (α = 0.1).

**Gene name and function**	**Gene symbol**	**Log_2 _ratio**	**GeneBank Acc. No.**
*Cytochrome P450 superfamily – xenobiotic metabolism*			
Cytochrome P450, 1a2	Cyp1a2	0.57*	NM_009993
Cytochrome P450, 2a4	Cyp2a4	1.89*	BC011233
**Cytochrome P450, 2b10**	**Cyp2b10**	9.14*	AK028103
**Cytochrome P450, 2b13**	**Cyp2b13**	9.94*	NM_007813
Cytochrome P450, 2c40	Cyp2c40	0.79	NM_010004
Cytochrome P450, 2f2	Cyp2f2	-0.54*	NM_007817
Cytochrome P450, 3a25	Cyp3a25	2.09*	BC028855
**Cytochrome P450, 3a41**	**Cyp3a41**	3.24*	NM_017396
*Cytochrome P450 superfamily – endogene metabolism*			
Retionic acid hydrolase	Cyp26a1	-0.72*	NM_007811
Thromboxane A synthase 1	Cyp5a1	-1.11	NM_011539
Cytochrome P450, 7b1	Cyp7b1	-0.88	BC038810
*Nuclear receptors superfamily*			
Constitutive androstane receptor	Nr1i3	3.51*	NM_009803
Testis receptor	Nr2c1	-0.67*	NM_011629
Glucocorticoid receptor	Nr3c1	1.84*	NM_008173
*Transporters*			
Ileal sodium-dependent bile acid transporter	Slc10a2	-1.55*	NM_011388
*Heme metabolism*			
**Delta-aminolevulinate synthase 1**	**Alas1**	3.36*	NM_020559

Genes in bold – RT-PCR analysis is consistent with microarray data.

*α = 0.05. Cyp3a41 and Cyp2b13 probes cross-hybridize with Cyp3a11 and Cyp2b10 respectively.

Note. Three mice were used in this experiment.

In cholesterol feeding experiment, we detected down-regulation of the cholesterol biosynthesis (*Fdft1*, *Sqle*, *Cyp51a1*, *Nsdhl*) on both platforms (Table 4 and Additional file 7). However, with Sterolgene we additionally detected up-regulation of the following genes: drug-metabolizing enzyme gene *Cyp3a11 *and nuclear receptor *Shp *(*Nr0b2*, small heterodimeric partner) as has been previously shown [23,24], cholesterol transporter *Scp2 *(Sterol carrier protein 2), and *Ldlrap1 *(LDL receptor adaptor protein 1). *Cyp3a11 *and *Fdft1 *were confirmed by RT-PCR (Figure 4D).

**Table 4:** Differentially expressed genes in the mouse liver after cholesterol feeding (Sterolgene v0 microarray). Differentially expressed genes in the mouse liver after cholesterol feeding as detected by the Sterolgene array (α = 0.1).

**Gene name and function**	**Gene symbol**	**Log_2 _ratio**	**GeneBank Acc. No.**
*Cholesterol biosynthesis*			
Acetyl-Coenzyme A synthetase 2	Acas2	-2.35	NM_019811
Phosphomevalonate kinase	Pmvk	-1.18	NM_026784
**Farnesyl diphosphate farnesyl transferase 1**	**Fdft1**	**-2.46**	NM_010191
Squalene epoxidase	Sqle	-2.47*	BC042781
Lanosterol 14α-demethylase	Cyp51a1	-1.90	BC031813
NAD(P) dependent steroid dehydrogenase-like	Nsdhl	-2.06*	BC019945
*Bile acids biosynthesis*			
Cholesterol 7α-hydroxylase	Cyp7a1	-1.44*	NM_007824
Cytochrome P450, 7b1	Cyp7b1	-2.01*	BC038810
*Cholesterol transport*			
LDL receptor adaptor protein 1	Ldlrap1	0.76	NM_145554
Sterol carrier protein 2	Scp2	1.09*	BC018384
*Nuclear receptor superfamily*			
Small heterodimeric partner	Nr0b2	0.94*	NM_011850
Testis receptor	Nr2c1	0.60*	NM_011629
Glucocorticoid receptor	Nr3c1	0.83*	NM_008173
*Cytochrome P450 superfamily – xenobiotic metabolism*			
Cytochrome P450, 2g1	Cyp2g1	1.09	NM_013809
**Cytochrome P450, 3a41**	**Cyp3a41**	**1.92**	NM_017396

Genes in bold – RT-PCR analysis is consistent with microarray data.

*α = 0.05. Cyp3a41 probe cross-hybridizes with Cyp3a11.

Note. Two mice were used in this experiment.

Additionally, we have selected only genes that are present in both platforms (we found 92 such genes) and determined their differential expression using the same p-value cut-off as for Sterolgene data (α = 0.1), with the same rationale as for the analysis of Agilent data. In phenobarbital experiment we found 21 genes differentially expressed (Additional file 8) and of these *Alas1 *and *Cyp2b10 *were confirmed by Sterolgene and RT-PCR, *Cyp2a4 *and *Nr2c1 *by Sterolgene (RT-PCR not performed), *Scap *by RT-PCR, while for *Apoa1 *and *Scarb1 *no confirmation was found (Figure 4C).

In cholesterol feeding experiment we found 41 genes differentially expressed (Additional file 9) and of these *Fdft1 *was confirmed by Sterolgene and RT-PCR, *Acss2*, *Cyp51a1*, *Nsdhl*, *Pmvk *and *Sqle *by Sterolgene (RT-PCR not performed), *Cyp26a1 *by RT-PCR, while for *Ppara *and *Scap *no confirmation was found (Figure 4D).

From the 92 genes that are present in both platforms we selected those that showed signal on both platforms and calculated a Pearson's product moment correlation coefficient between log_2 _ratios for data from both experiments. Figure 5B shows scatterplot of that data. We observed a similar correlation as for the Agilent-Steroltalk comparison (r = 0.499, p = 0.000, N = 319) and comparable to correlations reported by other cross-platform comparison studies [12,17-19]. Since more probes per gene are available on Affymetrix array we included all in the correlation analysis.

## Discussion

Systemic studies of cholesterol homeostasis and drug metabolism are still limited. In order to promote the implementation of systems biology approaches to this two research areas, we have developed the Sterolgene array. It is a highly customizable tool that together with its human version allows for detailed studies of the two biological processes in mouse and in human. The main goal of this study was to evaluate the performance of the Sterolgene array in typical studies of cholesterol homeostasis and drug metabolism in comparison with two commercial platforms (Agilent and Affymetrix). Several recent papers discuss the comparative data between different microarrays platforms [12,17-19,25]. Many of these studies calculated different correlation coefficients, measured reproducibility, but some have also taken into consideration the biological aspects of the comparison [12,19,26]. This approach was also undertaken in our evaluations of the performance of the Sterolgene array.

**Figure 6:**
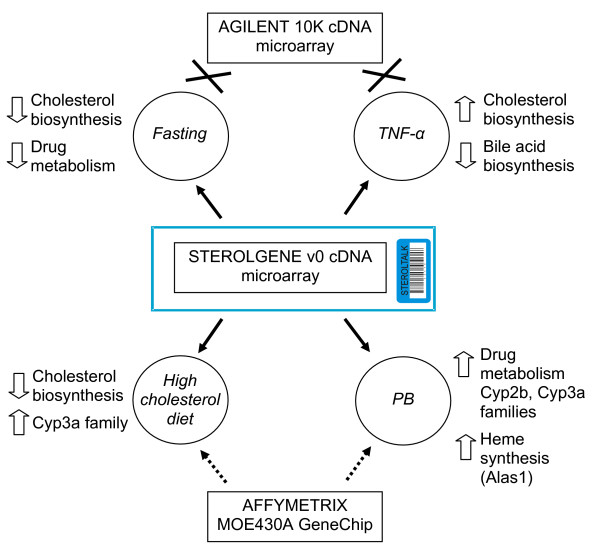
**Changes in cholesterol homeostasis and drug metabolism caused by different factors in mouse liver**. With Sterolgene array we successfully detected all changes in cholesterol homeostasis and drug metabolism caused by high-cholesterol diet, fasting, TNF-α, and phenobarbital (PB) treatment (solid arrows). With Agilent array (G4104A) we detected none of the changes caused by the inflammatory cytokine TNF-α and fasting (crosses). With Affymetrix MOE430A GeneChip we detected only down-regulation of the cholesterol biosynthesis by the high-cholesterol diet and induction of the *Cyp2b *family by the phenobarbital treatment (dashed arrows), but not the up-regulation of C*yp3a11 *by high-cholesterol diet and up-regulation of *Cyp3a *family and *Alas1 *by phenobarbital treatment. In all experiments, two tailed Student's t-test was used for classification of differentially expressed genes.

We have selected different factors, such as diet, drug treatment and inflammation, where scattered information existed about their effect on cholesterol homeostasis and drug metabolism (Figure 6). We analyzed the data as it is commonly done in microarray experiments. In the experiment where mice were on a high-cholesterol diet, we detected genes from cholesterol biosynthesis as down-regulated using both Sterolgene and Affymetrix arrays. However, with Sterolgene arrays, but not with Affymetrix, we detected *Cyp3a *family and nuclear receptor *Nr0b2 *as up-regulated and as previously described in [23,24]. In the liver of mice treated with phenobarbital, we detected up-regulation of the *Cyp2b *family using both Sterolgene and Affymetrix arrays and as previously described in [21]. However, with Sterolgene arrays we additionally detect genes from the *Cyp3a *family, *Cyp2a4 *and *Alas1 *as up-regulated and as previously described in [20,22]. Analysis of Affymetrix data using the same p-value cut-off as for the Sterolgene data showed two additional genes (*Abca1*, *Abcg5*) as differentially expressed in cholesterol feeding experiment, which were confirmed by another study [24] where also Affymetrix arrays were used. However, we were sill not able to confirm the up-regulation of *Cyp3a11 *and *Nr0b2*. In phenobarbital experiment we were able to confirm the up-regulation of *Alas1 *and *Cyp2a4*, but not *Cyp3a11*.

Low agreement was observed between the Agilent and the Sterolgene arrays in detecting changes in gene expression, regardless of the selected p-value cut-off. In fasting and TNF-α/fasted experiments we were able to detect changes in expression of genes related to cholesterol homeostasis and drug metabolism with Sterolgene arrays, but not with Agilent arrays. In fasting experiment, we observed metabolic adaptations to energy-shortage. Energy-consuming processes as are cholesterol biosynthesis and drug metabolism are down-regulated. However, treatment with TNF-α in combination with fasting reverses these adaptations and up-regulation of cholesterol biosynthesis and down-regulation of bile acids synthesis is observed. This results in disrupted cholesterol homeostasis and higher levels of cholesterol present in the mouse liver. All these findings are in agreement with the hypothesis that pro-inflammatory factors such as TNF-α are atherogenic [27].

Cross-platform comparisons using correlation analysis showed correlations comparable to other cross-platform comparison studies [12,17-19]. This result, together with low correspondence in differential expression (especially observed in Sterolgene-Agilent comparison) provides strong justification for development of small-scale custom arrays. As it turned out with Sterolgene data, small-scale arrays are often more sensitive than whole-genome arrays owing to the special care taken in designing probes.

Though we were able to show some important correspondence between the Steroltalk and both commercial platforms, our comparison has some limitations. First, we were limited by the availability of samples, resulting in low number of biological replicas used for comparisons. Additional samples and other diets/treatments may provide valuable information for the comparison and further insight into the performance of Sterolgene array. Second, we employed t-test based classification of differential expression as this is usually the case in most biologically-oriented studies. This may result in unstable variance (noise) estimation [26]. Other less rigid classification methods were developed, especially for microarray data analysis where we are usually dealing with small number of measurements. Although such methods might result in better correspondence between the platforms, we still decided to use a statistically-based method in order to avoid the biases that alternative approaches often introduce into results. Third, lab/platform effect, which can often be higher than the biological effect [12,18,28,29] must not be disregarded in the Sterolgene-Affymetrix comparison. And most importantly, gene probes are not sequence matched across the compared platforms, which in combination with different approaches to probe design and transcript variant detection can make a major contribution to low agreement [30,31]. Finally, labeling step and the choice of using amplification also contribute to differences [32,33].

One of the obstacles in conducting the comparison, but not the reason for low concordance, was the lack of annotation for probes on the commercial platforms. With all the tools available for microarray annotation there is still a large portion of probes whose annotation remain unknown and need a one by one search in the public databases. Well annotated low density microarrays, such as is Sterolgene, facilitate less demanding biological interpretation and confirmation of results. Another problem exposed in early papers on cDNA microarrays is also a high rate of incorrect clones in the cDNA libraries used for their production [17,34]. For Sterolgene array we have prepared our own cDNA library which we confirmed by sequencing.

An important advantage of small arrays is in the design of the probes. For whole-genome arrays, probes are designed (semi)automatically with a limited attention dedicated to each gene probe. With smaller arrays, more effort and *a priori *knowledge is invested into their design, thus enabling more accurate detection of changes in gene expression. The costs of producing and using small-scale academic arrays are still low compared to commercial ones. This is especially true for studies where a large number of microarray experiments is planned, e.g. screening process in the development of new drugs.

## Conclusion

We have demonstrated that the Sterolgene array is a novel tool in studies of cholesterol homeostasis and drug metabolism and can be used for more focused and cost-effective systemic studies. Limited to our studies, we have shown that its performance is comparable to the two commercial platforms. Its application ranges from basic research of how different factors influence these two biological processes to testing effects of new cholesterol lowering drugs. It is a tool which can be easily adjusted to novel research needs and interests. We are constantly improving the array with new genes and probes; we have recently developed the third version of the array, which contains an expanded list of genes involved in atherogenesis, circadian rhythm, inflammation and metabolism of carbohydrates and lipids, together with its human version consisting of human counterparts of most of the genes, thus allowing analysis of clinical samples.

## Methods

### Chemicals

All chemicals were purchased from Sigma (St Louis, MI, USA) if not stated otherwise.

### Mouse experiments and sample preparation

In the TNF-α (tumor necrosis factor alpha) and fasting experiments male mice C57BL/6 (Harlan, Italy) age between 10 and 12 weeks were housed in normal light-cycle room, maintained on standard rodent chow, and were allowed water and food *ad libitum *[15]. Three groups of mice, 5–8 animals per group, were injected *i.v *at the same time point either with vehicle (saline) or with human recombinant TNF-α 30 μg/animal in saline (a gift from V. Menart, Lek Pharmaceuticals d.d.) and food was withdrawn, except for the control group. After 20 h mice were sacrificed using CO_2 _suffocation. Total RNA was isolated from liver using TRI-reagent (Sigma, St Louis, MI, USA) and purified by RNeasy Mini Kit (Qiagen GmBH, Hilden, Germany) according to the manufacturer's protocols.

In phenobarbital treatment and cholesterol feeding experiments male mice C57BL/6 (RCC, Ittingen, Switzerland) age between 9 and 11 weeks were maintained in a 12-hour light/12-hour dark cycle and fed *ad libitum *standard rodent chow diet or the same diet supplemented with 1% (w/w) cholesterol (Provimi Kliba SA, Kaiseraugst, CH). Mice were divided in three groups, 8 animals per group: the control group which was injected *i.p. *vehicle (5% DMSO-corn oil) and maintained on standard diet; the cholesterol group which was maintained on cholesterol diet for week prior injection and injected *i.p. *vehicle; and the phenobarbital group which was maintained on standard diet and injected *i.p. *with 50 mg/kg phenobarbital sodium in a 5% DMSO-corn oil. After 10 hours, animals were sacrificed using CO_2 _suffocation. The livers were snap-frozen in liquid nitrogen, total RNA was isolated using TRIzol reagent (Invitrogen, Carlsbad, CA, USA) according to the manufacturer protocol and purified by RNeasy Mini Kit (Qiagen GmBH, Hilden, Germany). The total RNAs of two animals were pooled in equal amounts resulting in four pools per group. Quality and purity of total RNAs from both studies were checked using the RNA 6000 Nano LabChip Kit on Agilent 2100 Bioanalyzator (Agilent Technologies, Pato Alto, CA, USA). All animal experiments followed the Amsterdam Protocol on Animal Protection and Welfare and were approved by the local ethics committees.

### cDNA microarray production

For 95 selected genes cDNA microarray probes of a similar length (400 – 500 bp), melting temperature and located less than 1500 bp from the polyA tail were designed using ProbeWIZ software [35]. Three probes of firefly luciferase in sequential order from the 5'-end to the polyA tail have been designed as controls of the reverse transcriptase reaction and as normalization controls. Chloramphenicol acetyltransferase (CAT) and *Renilla *luciferase gene probes were designed as negative controls. For housekeeping genes two to three probes were designed enabling control of RNA degradation rate. PCR products were prepared and cloned using the Qiagen PCR Cloning kit (Qiagen GmBH, Hilden, Germany). Plasmids were sequenced to verify the correct insert sequence. From these plasmids PCR products were amplified (Qbiogene, Morgan Irvine, CA, USA) and purified using the QIAquick 96 PCR BioRobot purification kit (Qiagen GmBH, Hilden, Germany). PCR products were dried and dissolved in 50% formamide and 1% CHAPS (3-[(3-cholamidopropyl)-dimethylammonio]-1-propane sulfonate) spotting buffer in final 200 ng/μl concentration. DNAs were spotted on GAPS II-coated slides (Corning Inc. Corning, NY, USA) using a GeneTAC G3 robot (Genomic Solutions, Huntington, UK) in a controlled environment (25°C to 26°C and 25–29% humidity). Each PCR product was spotted in triplicates in two blocks yielding 6 replicas. Firefly luciferase probes were spotted in each block. Commercial "spike in controls" ArrayControl Spikes (Ambion, Austin, TX, USA) were also spotted, two in each block. After spotting slides were dried for 8 hours at room temperature, protected from light. The DNA was fixed by UV cross-linking at 250 mJ/cm^2 ^(Hoefer UVC500, Amersham Pharmacia Biotech, San Francisco, CA, USA). Slides were stored in a dry and dark place.

### Probe labeling and hybridizations

In TNF-α/fasted and fasting experiments, individual samples from fasted and TNF-α-treated groups were hybridized versus a reference pool of samples from normal-fed control group using Sterolgene (3 biological replicas) and Agilent arrays (2 biological replicas, identical to those used in Sterolgene hybridizations). The hybridizations were performed in a single laboratory according to the recommended protocols.

In phenobarbital and high cholesterol feeding experiments, samples from 8 mice were pooled by two into 4 pools for each control, cholesterol fed and phenobarbital treated group. All 4 pools from each group were hybridized to Affymetrix GeneChips. 2–3 pools, identical to those used in Affymetrix hybridizations, were hybridized together with a reference pool (pool of all samples) to Sterolgene arrays. Hybridizations of each of the platforms were performed in two different laboratories according to the recommended protocols.

In Sterolgene experiments, 10 μg of total RNA was used for labeling using Agilent Fluorescent Direct Label Kit (Agilent Technologies, Pato Alto, CA, USA) according to the manufacturer's protocol. Before labeling spike RNAs were added to the total RNA in following quantities: 500 pg of Firefly luciferase mRNA (Promega, Madison, WI, USA); and one of the two mixes (reference mix and sample mix) of 8 RNAs from ArrayControl Spikes (Ambion, Austin, TX, USA). The reference spike mix was a mixture of 500 pg of each RNA. The sample spike mix was a mixture of RNAs ranging from 100 pg to 1500 pg. In the TNF-α and fasting experiment, the control group was labeled with Cy3 (PerkinElmer Life Sciences, Boston, MA, USA), the fasted and the TNF-α treated groups were labeled with Cy5 (PerkinElmer Life Sciences, Boston, MA, USA). In the phenobarbital and cholesterol experiment the control, cholesterol and phenobarbital groups were labeled with Cy5 and the reference was labeled with Cy3. Sterolgene slides were washed before hybridization at room temperature for 5 min in 2 × SSC and 0.1%SDS, 3 min in 0.2 × SSC and 2 min in MilliQ water and dried by centrifugation. Samples were denatured and hybridized in 3 × SSC and 0.2% SDS hybridization buffer at 65°C for 16 hours using humidified hybridization chambers (HybChambers, GeneMachines, San Carlos, CA, USA). After hybridization slides were washed at room temperature for 5 min in 2 × SSC and 0.5% SDS, followed by 5 min in 0.5 × SSC, then 5 min in 0.05 × SSC and dried by centrifugation. Slides were scanned using a Tecan LS200 scanner (Tecan Group Ltd., Maennedorf, Switzerland).

In Agilent experiments, 10 μg of total RNA was used for labeling by Agilent Fluorescent Direct Label Kit (Agilent Technologies, Pato Alto, CA, USA). Preparation of samples, hybridization and washing protocols were done according to Mouse cDNA Microarray Kit (Agilent Technologies, Santa Clara, CA, USA) using the mouse Agilent cDNA microarrays (G4104A). The control group was labeled with Cy3, the fasted and the TNF-α treated group were labeled with Cy5. Hybridizations were performed using hybridization chambers (HybChambers, GeneMachines, San Carlos, CA, USA) and slides scanned by Tecan LS200 scanner (Tecan Group Ltd., Maennedorf, Switzerland).

In Affymetrix experiments, total RNA was analyzed by the MOE430A GeneChip (Affymetrix, Santa Clara, CA, USA) according to manufacturer's recommendations and slides scanned by a laser scanner (Affymetrix, Santa Clara, CA, USA) as described in [36].

### Data normalization and analysis

Images of Sterolgene arrays were analyzed by Array-Pro Analyzer 4.5 (Media Cybernetics, Bethesda, MD, USA). The median feature and local background intensities were extracted together with the estimates of their standard deviation. Only features with foreground to background ratio higher than 1.5 and coefficient of variation (CV, ratio between standard deviation of the background and the median feature intensity) lower than 0.5 in both channels were used for further analysis. Log_2 _ratios were normalized using LOWESS fit [37] to spike in control RNAs according to their average intensity. For normalization, two types of spike in controls were used: custom-made Firefly luciferase (348 such probes per array) and commercial ArrayControl Spikes (Ambion, Austin, TX, USA) (96 such probes per array). Ambion spike in RNAs were added to each sample RNAs in the following concentrations: 1:5, 1:2, 1:1, 2:1 and 3:1. Data from Ambion spike in controls were adjusted prior to fit of normalization curve by subtracting log_2_(1/5), log_2_(1/2), log_2_(2) and log_2_(3) from the log_2 _ratios of measured intensities according to the concentrations (Figure 3C). In phenobarbital and cholesterol-feeding experiments data were standardized (median-centered and scaled by median absolute deviation) in order to reduce inter-array variability. Mean was used to average replicated microarray measurements. Data normalization and standardization was done in Orange software [13].

Images of Agilent microarrays were analyzed using Array-Pro Analyzer 4.5 (Media Cybernetics, Bethesda, MD, USA). Features, with CV > 0.39, were filtered out and data were normalized using LOWESS fit to all genes according to their average intensity [38]. Filtration and normalization was done in BASE software [39].

Affymetrix data were normalized by the Robust Multichip Average (RMA) algorithm as described [40]. After transformation to non-logarithmic data, the expression estimates were scaled to the average expression levels in the control group analyzed using GeneSpring software (Silicon Genetics, Redwood, USA).

Classification of differentially expressed genes was done in Orange using two tailed Student's t-test for independent samples. Between the platforms genes were matched by unigene or refseq codes, or by gene symbols. Pearson's product moment correlation coefficients were calculated and scatterplots produced in SPSS 14.0. All data have been submitted to GEO (Gene expression omnibus) under accession codes: GSE6271 (Affymetrix data), GSE6317 (Agilent data), GSE6447 (Sterolgene phenobarbital and high-cholesterol diet data), and GSE6423 (Sterolgene fasting and inflammation data).

### Real-time polymerase chain reaction (RT-PCR)

RT-PCR analyses were performed in triplicates on pools of 3 mice from each group in TNF-α and fasting experiments, and on 4 biological replicas in phenobarbital and high-cholesterol diet experiments. In TNF-α and fasting experiments 1 μg of total RNA was reverse-transcribed using Superscript™ II or III reverse transcriptase (Invitrogen, Carlsbad, CA, USA). Analyses were done using Platinum^® ^SYBR^® ^Green qPCR SuperMix-UDG (Invitrogen, Carlsbad, CA, USA), except in case of *Sc4mol *where Assay-on-demand from Applied Biosystems was used, and were performed on Applied Biosystems ABI PRISM 7900 HT or 7500 (PE Applied Biosystems, Foster City, CA, USA) according to the manufacturer's protocol. In phenobarbital and high-cholesterol diet experiments 1 μg of total RNA was reverse-transcribed using MMLV reverse transcriptase (Roche Molecular Biochemicals, Rotkreuz, Switzerland). Analyses (for transcripts *Cyp3a11, Cyp2b10, Alas1*) were done using the TaqMan PCR Core Reagent Kit (PE Applied Biosystems, Rotkreuz, Switzerland), and performed on ABI PRISM 7700 Sequence Detection System (PE Applied Biosystems, Rotkreuz, Switzerland) according to the manufacturer's protocol. Additional analyses (for transcript *Scap*, *Apoa1*, *Car*, *Cyp7a1*, *Cyp1a2*, *Scarb1*, *Ppara*, *Cyp26a1*) were done using Platinum^® ^SYBR^® ^Green qPCR SuperMix-UDG (Invitrogen, Carlsbad, CA, USA) and 7500 (PE Applied Biosystems, Foster City, CA, USA) according to the manufacturer's protocol. Relative transcript levels were determined using the comparative Ct (cycle threshold) method or using 2^-ddCt ^values [41]. Internal control used in the first set of experiments is 18s and in the second set *Gapdh*. Statistical significance was determined by two tailed Student's t-test (α = 0.05). Primer sequences are provided in additional material (Additional file 10).

## Authors' contributions

TR contributed to the gene list, probe and overall microarray design, and performed cloning and preparations of probes for microarray manufacturing. TR also designed protocols for Sterolgene analysis, performed hybridizations and part of the statistical analyses. PJ developed normalization and analysis tools and performed part of the statistical analysis. KFT performed TNF-α and fasting mouse experiments, and analyzed samples using Agilent and Sterolgene microarrays. DK contributed to probe design and microarray manufacturing, and performed first bioinformatical analyses. AR and UAM contributed to the gene list and AR performed phenobarbital and high-cholesterol diet experiments on mouse and Affymetrix hybridizations under supervision of UAM. LPA cooperated in microarray production. DP contributed to the gene list and concept of Sterolgene microarrays and was involved in manufacturing process. DR contributed to the gene list and concept of Sterolgene microarrays and was a supervisor of TR and KFT. All authors have read and approved the final manuscript.

## Supplementary Material

Additional file 1 List of genes present on the Sterolgene v0 cDNA microarray Click here for file

Additional file 2 **Differentially expressed genes in the mouse liver after TNF-α treatment (Agilent microarray)**. Differentially expressed genes in the mouse liver after TNF-α treatment as detected by the Agilent 10 K cDNA microarray (G4104A) (α = 0.01, genes in bold: α = 0.001). Click here for file

Additional file 3 **Differentially expressed genes in the mouse liver after fasting (Agilent microarray)**. Differentially expressed genes in the mouse liver after fasting as detected by the Agilent cDNA microarray (G4104A) (α = 0.01, genes in bold: α = 0.001). Click here for file

Additional file 4 **Differentially expressed genes in mouse liver after fasting using the same probability of type I error as for Sterolgene array (Agilent microarray)**. Differentially expressed genes as detected by Agilent 10 K cDNA microarray using the same probability of type I error as for Sterolgene data (α = 0.05). Only genes that are also present in the Steroltalk array were considered in the analysis. Genes in bold are confirmed using RT-PCR, genes in italic coincide with the results from the Sterolgene platform. Click here for file

Additional file 5 **Differentially expressed genes in mouse liver after TNF-α treatment using the same probability of type I error as for Sterolgene array (Agilent microarray)**. Differentially expressed genes as detected by Agilent 10 K cDNA microarray using the same probability of type I error as for Sterolgene data (α = 0.05). Only genes that are also present in the Steroltalk array were considered in the analysis. Genes in bold are confirmed using RT-PCR, genes in italic coincide with the results from the Sterolgene platform. Click here for file

Additional file 6 **Differentially expressed genes in the mouse liver after phenobarbital treatment (Affymetrix GeneChip)**. Differentially expressed genes in the mouse liver after phenobarbital treatment as detected by the Affymetrix MOE430A GeneChip (α = 0.001, genes in bold: α = 0.00043). Click here for file

Additional file 7 **Differentially expressed genes in the mouse liver after cholesterol feeding (Affymetrix GeneChip)**. Differentially expressed genes in the mouse liver after cholesterol feeding as detected by the Affymetrix MOE430A GeneChip (α = 0.001, genes in bold: α = 0.00043). Click here for file

Additional file 8 **Differentially expressed genes in mouse liver after phenobarbital treatment using the same probability of type I error as for Sterolgene array (Affymetrix GeneChip)**. Differentially expressed genes as detected by Affymetrix GeneChip using the same probability of type I error as for Sterolgene data (α = 0.1). Only genes that are also present in the Steroltalk array were considered in the analysis. Genes in bold are confirmed using RT-PCR, genes in italic coincide with the results from the Sterolgene platform. Click here for file

Additional file 9 **Differentially expressed genes in mouse liver after cholesterol feeding using the same probability of type I error as for Sterolgene array (Affymetrix GeneChip)**. Differentially expressed genes as detected by Affymetrix GeneChip using the same probability of type I error as for Sterolgene data (α = 0.1). Only genes that are also present in the Steroltalk array were considered in the analysis. Genes in bold are confirmed using RT-PCR, genes in italic coincide with the results from the Sterolgene platform. Click here for file

Additional file 10 Primer sequences used in quantitative RT-PCR analyses Click here for file
