# Supplementary material for: The Sterolgene v0 cDNA microarray: a systemic approach to studies of cholesterol homeostasis and drug metabolism
Source: BMC Genomics. 2008 Feb 11;9:76. doi: 10.1186/1471-2164-9-76 (PMC2262072; doi:10.1186/1471-2164-9-76)
Supplement: Additional file 1 — List of genes present on the Sterolgene v0 cDNA microarray [file 1471-2164-9-76-S1.pdf]

Gene name	Gene symbol	GeneBank Acc. No.
ABC transporter superfamily		
Cholesterol efflux regulatory protein	Abca1	NM_013454
Multidrug resistance 1a	Abcb1a	NM_011076
Multidrug resistance 1b	Abcb1b	NM_011075
Multidrug resistance 2	Abcb4	NM_008830
ABC transporter 7	Abcb7	BC035534
Multidrug resistance associated protein 2	Abcc2	NM_013806
Sterolin1	Abcg5	NM_031884
Cytochrome P450 superfamily		
Cytochrome P450, 1a1	Cyp1a1	NM_009992
Cytochrome P450, 1a2	Cyp1a2	NM_009993
Cholesterol 27-hydroxylase	Cyp27a1	NM_024264
Steroid 7α-hydroxylase	Cyp2a12	NM_133657
Cytochrome P450, 2a12	Cyp2a4	BC011233
Cytochrome, 2b10	Cyp2b10	AK028103
Cytochrome, 2b13	Cyp2b13	NM_007813
Cytochrome, 2c40	Cyp2c40	NM_010004
Cytochrome, 2d22	Cyp2d22	NM_019823
Cytochrome, 2f2	Cyp2f2	NM_007817
Cytochrome, 2g1	Cyp2g1	NM_013809
Cytochrome, 2j5	Cyp2j5	NM_010007
Cytochrome, 2j6	Cyp2j6	U62295
Cytochrome, 3a25	Cyp3a25	BC028855
Cytochrome, 3a41	Cyp3a41	NM_017396
Thromboxane A synthase 1	Cyp5a1	NM_011539
Cholesterol 7α-hydroxylase	Cyp7a1	NM_007824
Cytochrome, 7b1	Cyp7b1	BC038810
Prostacyclin synthase	Cyp8a1	NM_008968
Sterol 12α-hydroxylase	Cyp8b1	NM_010012
Cholesterol side chain cleavage enzyme	Cyp11a1	NM_019779
Steroid 11β-hydroxylase	Cyp11b2	NM_009991
Steroid 17α-hydroxylase	Cyp17a1	NM_007809
Cytochrome, 20a1	Cyp20a1	BC049147
Retionic acid hydrolase	Cyp26a1	NM_007811
Cholesterol 24-hydroxylase	Cyp46a1	NM_010010
Lanosterol 14α-demethylase	Cyp51a1	BC031813
Cholesterol biosynthesis		
Acetyl-Coenzyme A synthetase 2	Acas2	NM_019811
Acetyl-CoA acetyltransferase 2	Acat2	NM_009338
Mevalonate kinase	Mvk	NM_023556
Phosphomevalonate kinase	Pmvk	NM_026784
Mevalonate (diphospho) decarboxylase	Mvd	NM_138656
Farnesyl diphosphate synthase 1	Fdps	NM_134469
Farnesyl diphosphate farnesyl transferase 1	Fdft1	NM_010191
Squalene epoxidase	Sqle	BC042781

Lanosterol synthase	Lss	NM_146006
24-dehydrocholesterol reductase	Dhcr24	BC019797
Sterol-C4-methyl oxidase-like	Sc4mol	NM_025436
NAD(P) dependent steroid dehydrogenase-like	Nsdhl	BC019945
Emopamil binding protein	Ebp	NM_007898
Sterol C5 desaturase	Sc5d	BC024132
7-dehydrocholesterol reductase	Dhcr7	BC006854
Nuclear receptor superfamily		
Small heterodimeric partner	Nr0b2	NM_011850
Thyroid hormone receptor alpha	Nr1a1	NM_178060
Thyroid hormone receptor beta	Nr1a2	NM_009380
Retinoic acid receptor alpha	Nr1b1	NM_009024
Peroxisome proliferator-activated receptor alpha	Nr1c1	NM_011144
Peroxisome proliferator-activated receptor gamma	Nr1c3	NM_011146
Reverse ErbA beta	Nr1d2	NM_011584
Retinoic acid-related orphan receptor alpha	Nr1f1	NM_013646
Retinoic acid-related orphan receptor gamma	Nr1f3	NM_011281
Liver X receptor beta	Nr1h2	NM_009473
Farnesoid X receptor	Nr1h4	NM_009108
Vitamin D receptor	Nr1i1	NM_009504
Constitutive androstane receptor	Nr1i3	NM_009803
Hepatocyte nuclear factor 4	Nr2a1	NM_008261
Retinoid X receptor alpha	Nr2b1	NM_011305
Retinoid X receptor beta	Nr2b2	NM_011306
Retinoid X receptor gamma	Nr2b3	NM_009107
Testis receptor	Nr2c1	NM_011629
Tailles-related receptor	Nr2e1	NM_152229
Chicken ovalbumin upstream promoter - transcription factor	Nr2f2	NM_009697
ErbA-related 2	Nr2f6	NM_010150
Estrogen related receptor alpha	Nr3b1	NM_007953
Estrogen related receptor beta	Nr3b2	NM_011934
Glucocorticoid receptor	Nr3c1	NM_008173
Androgen receptor	Nr3c4	M37890
Thyroid hormone receptor	Nr4a1	NM_010444
Embryonal long terminal repeat-binding protein	Nr5a1	NM_139051
Liver receptor homologous protein 1	Nr5a2	NM_030676
Retinoid receptor-related testis-associated receptor	Nr6a1	AF390896
Cholesterol plasma transport		
Apolipoprotein A1	Apoa1	NM_009692
LDL receptor adaptor protein 1	Ldlrap1	NM_145554
Other transporters		
Scavenger receptor B1	Scarb1	NM_016741
Sterol carrier protein 2	Scp2	BC018384
Ileal sodium-dependent bile acid transporter	Slc10a2	NM_011388

	Organic anion transporter 1	Slco1a1	AY195868
Transcription factors			
	Sterol regulatory element binding transcription factor 1	Srebf1	NM_011480
	Sterol regulatory element binding transcription factor 2	Srebf2	NM_033218
	Sterol cleavage activating protein	Scap	NM_001001144
	CCAAT/enhancer binding protein alpha	Cebpa	BC028890
	PPARG coactivator 1	Ppargc1	NM_008904
	Transcription factor 1	Tcf1	NM_009327
	Steroidogenic acute regulatory protein	Star	AK054470
Heme synthesis			
	Delta-aminolevulinate synthase 1	Alas1	NM_020559
Housekeeping genes			
	Glyceraldehyde-3-phosphate dehydrogenase	Gapd	NM_008084
	Peptidylprolyl isomerase A	Ppia	NM_008907
