# Supplementary material for: The Sterolgene v0 cDNA microarray: a systemic approach to studies of cholesterol homeostasis and drug metabolism
Source: BMC Genomics. 2008 Feb 11;9:76. doi: 10.1186/1471-2164-9-76 (PMC2262072; doi:10.1186/1471-2164-9-76)
Supplement: Additional file 2 — Differentially expressed genes in the mouse liver after TNF-α treatment (Agilent microarray). Differentially expressed genes in the mouse liver after TNF-α treatment as detected by the Agilent 10 K cDNA microarray (G4104A) (α = 0.01, genes in bold: α = 0.001). [file 1471-2164-9-76-S2.pdf]

Log_2 ratio	Gene name	Gene Symbol	GeneBank Acc. No.
-3.18	cytochrome P450, family 4, subfamily a, polypeptide 10	Cyp4a10	AA755385
-2.72	cytochrome P450, family 2, subfamily f, polypeptide 2	Cyp2f2	AA220582
-2.48	coproporphyrinogen oxidase	Cpox	AA108600
-2.29	retinol binding protein 4, plasma	Rbp4	AA967219
-2.18	cytochrome P450, family 2, subfamily c, polypeptide 29	Cyp2c29	AA106162
-1.79	urate oxidase	Uox	AI551358
-1.56	fumarylacetoacetate hydrolase	Fah	AI180932
-1.52	solute carrier family 27 (fatty acid transporter), member 2	Slc27a2	AA066694
**-1.49**	**pipecolic acid oxidase**	**Pipox**	**AA242349**
-1.4	S-adenosylhomocysteine hydrolase	Ahcy	AA638891
-1.36	aldehyde dehydrogenase family 7, member A1	Aldh7a1	AA617418
-1.3	Transcribed locus	?	AA435317
-1.27	epoxide hydrolase 1, microsomal	Ephx1	AA822067
-1.15	glutathione S-transferase, theta 1	Gstt1	W54467
-1.1	D-dopachrome tautomerase	Ddt	AA638944
-1.08	integrin alpha 7	Itga7	AA062457
**-0.87**	**bile acid-Coenzyme A: amino acid N-acyltransferase**	**Baat**	**AA244588**
-0.86	4-aminobutyrate aminotransferase	Abat	AA261489
-0.84	ADP-ribosylation factor-like 6 interacting protein 2	Arl6ip2	AA414388
-0.83	tryptophan 2,3-dioxygenase	Tdo2	AA572623
-0.79	suppressor of initiator codon mutations, related sequence 1 (S. cerevisiae)	Sui1-rs1	AI182724
-0.79	progesterone receptor membrane component 1	Pgrmc1	AA117455
**-0.55**	**chaperonin subunit 5 (epsilon)**	**Cct5**	**AI325795**
-0.47	RIKEN cDNA 2400006N03 gene	2400006N03Rik	AA185481
-0.45	cell death-inducing DNA fragmentation factor, alpha subunit-like effector B	Cideb	AA290390
-0.38	RIKEN cDNA G431001E03 gene	G431001E03Rik	AA683963
-0.36	oxysterol binding protein-like 1A	Osbpl1a	AA414046
-0.1	mitochondrial ribosomal protein L17	Mrpl17	AA048585
0.25	gene rich cluster, C2f gene	Grcc2f	W62577
0.4	lecithin cholesterol acyltransferase	Lcat	AA250068
**0.44**	**Ribosomal protein L36a**	**Rpl36a**	**AA600581**
0.45	methyltransferase-like 1	Mettl1	AA770911
0.48	guanine nucleotide binding protein, beta 2, related sequence 1	Gnb2-rs1	AA571755
0.51	ribosomal protein S24	Rps24	AI892534
0.54	Ribosomal protein L27a	Rpl27a	AA822609
**0.66**	**RIKEN cDNA 2810418N01 gene**	**2810418N01Rik**	**AA543968**

0.8	Ribosomal protein L41	Rpl41	AI120332
1.27	actin, beta, cytoplasmic	Actb	AI594289
1.45	Sec61 beta subunit	Sec61b	AA638788
1.61	lymphocyte antigen 6 complex, locus E	Ly6e	AI326722
1.64	superoxide dismutase 2, mitochondrial	Sod2	AA415267
1.78	crystallin, mu	Crym	AA733693
**3.57**	**hemopexin**	**Hpxn**	**AA822009**
4.89	orosomucoid 1	Orm1	AI117779
4.97	serum amyloid P-component	Apcs	AA261401
5.09	orosomucoid 2	Orm2	AA245687
