# Supplementary material for: The Sterolgene v0 cDNA microarray: a systemic approach to studies of cholesterol homeostasis and drug metabolism
Source: BMC Genomics. 2008 Feb 11;9:76. doi: 10.1186/1471-2164-9-76 (PMC2262072; doi:10.1186/1471-2164-9-76)
Supplement: Additional file 3 — Differentially expressed genes in the mouse liver after fasting (Agilent microarray). Differentially expressed genes in the mouse liver after fasting as detected by the Agilent cDNA microarray (G4104A) (α = 0.01, genes in bold: α = 0.001). [file 1471-2164-9-76-S3.pdf]

Log_2 ratio	Gene name	Gene Symbol	GeneBank Acc. No.
-1.93	cytochrome P450, family 2, subfamily c, polypeptide 29	Cyp2c29	AA106162
-1.41	Histocompatibility 2, K1, K region	H2-K1	AA122791
-1.33	urate oxidase	Uox	AI551358
-1.3	hemopexin	Hpxn	AA822009
-1.11	serine (or cysteine) proteinase inhibitor, clade F, member 2	Serpinf2	W33918
-1.05	similar to Cytochrome P450, family 2, subfamily a, polypeptide 12	LOC233005	AA238327
-0.84	dehydrogenase/reductase (SDR family) member 3	Dhrs3	AA881922
-0.76	hydroxysteroid (17-beta) dehydrogenase 2	Hsd17b2	AA027607
-0.6	serine (or cysteine) proteinase inhibitor, clade A, member 1e	Serpina1e	W13979
-0.54	coagulation factor XII (Hageman factor)	F12	W34349
-0.46	lectin, galactose binding, soluble 9	Lgals9	AA674445
**-0.25**	**eukaryotic translation elongation factor 1 gamma**	**Eef1g**	**AI549639**
-0.21	ATP synthase, H+ transporting mitochondrial F1 complex, beta subunit	Atp5b	W36948
-0.18	dihydrolipoamide dehydrogenase	Dld	AA245976
0.31	basic transcription factor 3	Btf3	AA688716
0.46	Ribosomal protein L36a	Rpl36a	AA600581
0.5	histidine triad nucleotide binding protein 1	Hint1	AA068901
1.13	RIKEN cDNA G431001E03 gene	G431001E03Rik	AA683963
