# Supplementary material for: The Sterolgene v0 cDNA microarray: a systemic approach to studies of cholesterol homeostasis and drug metabolism
Source: BMC Genomics. 2008 Feb 11;9:76. doi: 10.1186/1471-2164-9-76 (PMC2262072; doi:10.1186/1471-2164-9-76)
Supplement: Additional file 4 — Differentially expressed genes in mouse liver after fasting using the same probability of type I error as for Sterolgene array (Agilent microarray). Differentially expressed genes as detected by Agilent 10 K cDNA microarray using the same probability of type I error as for Sterolgene data (α = 0.05). Only genes that are also present in the Steroltalk array were considered in the analysis. Genes in bold are confirmed using RT-PCR, genes in italic coincide with the results from the Sterolgene platform. [file 1471-2164-9-76-S4.pdf]

**Log$_2$ ratio**	**Gene name**	**Gene symbol**	**GeneBank Acc.No.**
-1.52	***cytochrome P450, 1a2***	***Cyp1a2***	***AA242360***
-1.05	cytochrome P450, 2a12	Cyp2a12	AA238327
-0.91	cytochrome P450, 2f2	Cyp2f2	AA220582
**-0.34**	**actin, beta, cytoplasmic**	**Actb**	**AI594289**
0.23	apolipoprotein A-I	Apoa1	AA822098
