# Supplementary material for: The Sterolgene v0 cDNA microarray: a systemic approach to studies of cholesterol homeostasis and drug metabolism
Source: BMC Genomics. 2008 Feb 11;9:76. doi: 10.1186/1471-2164-9-76 (PMC2262072; doi:10.1186/1471-2164-9-76)
Supplement: Additional file 5 — Differentially expressed genes in mouse liver after TNF-α treatment using the same probability of type I error as for Sterolgene array (Agilent microarray). Differentially expressed genes as detected by Agilent 10 K cDNA microarray using the same probability of type I error as for Sterolgene data (α = 0.05). Only genes that are also present in the Steroltalk array were considered in the analysis. Genes in bold are confirmed using RT-PCR, genes in italic coincide with the results from the Sterolgene platform. [file 1471-2164-9-76-S5.pdf]

Log_2 ratio	Gene name	Gene symbol	GeneBank Acc. No.
-3.93	cytochrome P450, 3a11	Cyp3a11	AA647336
-2.72	*cytochrome P450,2f2*	*Cyp2f2*	*AA220582*
**-0.54**	**apolipoprotein A-I**	**Apoa1**	**AA822098**
**1.27**	**actin, beta, cytoplasmic**	**Actb**	**AI594289**
