# Supplementary material for: The Sterolgene v0 cDNA microarray: a systemic approach to studies of cholesterol homeostasis and drug metabolism
Source: BMC Genomics. 2008 Feb 11;9:76. doi: 10.1186/1471-2164-9-76 (PMC2262072; doi:10.1186/1471-2164-9-76)
Supplement: Additional file 6 — Differentially expressed genes in the mouse liver after phenobarbital treatment (Affymetrix GeneChip). Differentially expressed genes in the mouse liver after phenobarbital treatment as detected by the Affymetrix MOE430A GeneChip (α = 0.001, genes in bold: α = 0.00043). [file 1471-2164-9-76-S6.pdf]

Log$_2$ ratio	Gene name	Gene Symbol	GeneBank Acc. No.
-0.38	Transducin-like enhancer of split 6, homolog of Drosophila E(spl)	Tle6	NM_053254
-0.37	WD repeat domain 6	Wdr6	BB453609
-0.32	Scavenger receptor class B, member 1	Scarb1	NM_016741
-0.27	DNA-damage-inducible transcript 4-like	Ddit4l	AF335325
-0.27	GIPC PDZ domain containing family, member 3	Gipc3	AB074494
-0.24	Proprotein convertase subtilisin/kexin type 1 inhibitor	Pcsk1n	AF181560
**-0.24**	**Seryl-aminoacyl-tRNA synthetase 2**	**Sars2**	**NM_023637**
**-0.23**	**GPI anchor attachment protein 1**	**Gpaa1**	**BB550093**
-0.21	C-type lectin domain family 3, member b	Clec3b	NM_011606
**-0.20**	**Cbp/p300-interacting transactivator with Glu/Asp-rich carboxy-terminal domain 1**	**Cited1**	**U65091**
-0.19	Phytanoyl-CoA hydroxylase interacting protein-like	Phyhipl	AI267048
**-0.17**	**Cyclin-dependent kinase inhibitor 2C (p18, inhibits CDK4)**	**Cdkn2c**	**AI323293**
-0.16	ST6 (alpha-N-acetyl-neuraminyl-2,3-beta-galactosyl-1,3)-N-acetylgalactosaminide alpha-2,6-sialyltransferase 6	St6galnac6	BB028302
**-0.16**	**Peptidylglycine alpha-amidating monooxygenase COOH-terminal interactor**	**Pamci**	**BC028805**
**-0.14**	**Tuberous sclerosis 1**	**Tsc1**	**BG073522**
-0.14	Cyclin-dependent kinase inhibitor 1C (P57)	Cdkn1c	NM_009876
-0.13	RIKEN cDNA 1810029B16 gene	1810029B16Rik	BC016246
-0.13	Leucine rich repeat containing 56	Lrrc56	AW553222
-0.12	growth differentiation factor 3	Gdf3	NM_008108
-0.10	Ankyrin repeat domain 43	Ankrd43	BB428991
-0.05	RIKEN cDNA 1200020A08 gene	1200020A08Rik	BB775592
**0.09**	**Sno, strawberry notch homolog 1 (Drosophila)**	**Sbno1**	**BG076340**
0.12	UDP glucuronosyltransferase 2 family, polypeptide B5	Ugt2b5	NM_009467
0.12	Sjogren syndrome antigen B	Ssb	BM208153
0.13	RIKEN cDNA 2310061C15 gene	2310061C15Rik	AW045976
0.13	Centrosomal protein 70	Cep70	NM_023873
0.13	F-box and WD-40 domain protein 2	Fbxw2	AK009893
**0.14**		**unknown**	**AV309422**
0.14	Feminization 1 homolog a (C. elegans)	Fem1a	AK005041
0.15	N-acylsphingosine amidohydrolase 1	Asah1	NM_019734
0.17	Terf1 (TRF1)-interacting nuclear factor 2	Tinf2	AF214013

**0.18**	**Signal recognition particle receptor ('docking protein')**	**Srpr**	**BC021839**
0.20	SWI/SNF-related, matrix-associated actin-dependent regulator of chromatin, subfamily a, containing DEAD/H box 1`	Smarcad1	BG065193
**0.20**	**Vacuolar protein sorting 37C (yeast)**	**Vps37c**	**BB270615**
**0.22**	**Transcribed locus**		**AW987547**
0.22	Yip1 interacting factor homolog A (S. cerevisiae)	Yif1a	BC011117
0.23	Nucleotide binding protein 1	Nubp1	NM_011955
0.24	Intestinal cell kinase	Ick	NM_019987
0.25	Leucine rich repeat containing 54	Lrrc54	BB548458
0.26	Sno, strawberry notch homolog 1 (Drosophila)	Sbno1	BE947961
**0.27**	**RIKEN cDNA 2310008H04 gene**	**2310008H04Rik**	**BC026877**
**0.28**	**Rhomboid domain containing 2**	**Rhbdd2**	**BB233055**
0.29	Prospero-related homeobox 1	Prox1	NM_008937
0.30	Endothelial cell-specific adhesion molecule	Esam1	AF361882
0.30	FMS-like tyrosine kinase 4	Flt4	NM_008029
**0.31**	**S-adenosylmethionine decarboxylase 1**	**Amd1**	**NM_009665**
**0.31**	**Similar to G/T mismatch-specific thymine DNA glycosylase (C-JUN leucine zipper interactive protein JZA-3), transcript variant 2 (LOC624784), mRNA**		**BF318654**
0.32	sulfiredoxin 1 homolog	Srxn1	BI731645
0.32	Solute carrier family 35 (UDP-glucuronic acid/UDP-N-acetylgalactosamine dual transporter), member D1	Slc35d1	BB409668
0.33	Peroxisomal biogenesis factor 12	Pex12	NM_134025
0.33	Platelet-activating factor acetylhydrolase, isoform 1b, beta1 subunit	Pafah1b1	BG975639
**0.38**		**unknown**	**AV166873**
**0.42**	**Mortality factor 4 like 1**	**Morf4l1**	**AU042749**
0.43	Abhydrolase domain containing 4	Abhd4	NM_134076
**0.43**	**Plexin C1**	**Plxnc1**	**BB765457**
**0.43**	**Transmembrane protein 56**	**Tmem56**	**BB667728**
0.46	Growth arrest specific 5	Gas5	BI650268
0.47	Aldehyde dehydrogenase family 1, subfamily A1	Aldh1a1	NM_013467
0.50	Phosphatidic acid phosphatase 2a	Ppap2a	NM_008903
**0.55**	**Carboxylesterase 6**	**Ces6**	**NM_133960**
**0.55**	**Inhibitor of kappaB kinase gamma**	**Ikbkg**	**BB147462**
0.69	ATP-binding cassette, sub-family C (CFTR/MRP), member 3	Abcc3	AK006128
**0.76**	**Cytochrome P450, family 2, subfamily c, polypeptide 50**	**Cyp2c50**	**NM_134144**
0.77	UDP glucuronosyltransferase 2 family,	Ugt2b34	BC028826

	polypeptide B34		
**0.77**	**Cytochrome P450, family 2, subfamily c, polypeptide 29**	**Cyp2c29**	**NM_007815**
**0.84**	**Ectonucleoside triphosphate diphosphohydrolase 5**	**Entpd5**	**AV376291**
0.89	Aryl-hydrocarbon receptor	Ahr	NM_013464
**1.01**	**Cytochrome P450, family 2, subfamily c, polypeptide 50**	**Cyp2c50**	**NM_010001**
1.04	RIKEN cDNA 4931406C07 gene	4931406C07Rik	NM_133732
**1.17**	**Cytochrome P450**	**Cyp2c54**	**AI256046**
1.23	RIKEN cDNA 4931406C07 gene	4931406C07Rik	AK016432
**1.49**	**P450 (cytochrome) oxidoreductase**	**Por**	**NM_008898**
**1.58**	**Glutathione S-transferase, mu 1**	**Gstm1**	**J03953**
**3.72**	**Cytochrome P450, family 2, subfamily c, polypeptide 55**	**Cyp2c55**	**NM_028089**
**4.90**	**Cytochrome P450, family 2, subfamily b, polypeptide 10**	**Cyp2b10**	**AF128849**
**6.29**	**Cytochrome P450, family 2, subfamily b, polypeptide 10**	**Cyp2b10**	**NM_009998**
**6.75**	**Cytochrome P450, family 2, subfamily b, polypeptide 10**	**Cyp2b10**	**AF128849**
