# Supplementary material for: The Sterolgene v0 cDNA microarray: a systemic approach to studies of cholesterol homeostasis and drug metabolism
Source: BMC Genomics. 2008 Feb 11;9:76. doi: 10.1186/1471-2164-9-76 (PMC2262072; doi:10.1186/1471-2164-9-76)
Supplement: Additional file 7 — Differentially expressed genes in the mouse liver after cholesterol feeding (Affymetrix GeneChip). Differentially expressed genes in the mouse liver after cholesterol feeding as detected by the Affymetrix MOE430A GeneChip (α = 0.001, genes in bold: α = 0.00043). [file 1471-2164-9-76-S7.pdf]

Log$_2$ ratio	Gene Name	Gene Symbol	GeneBank Acc. No.
**-3.42**	**Adenosine deaminase, RNA-specific, B2**	**Adarb2**	**BC004801**
-3.35	Squalene epoxidase	Sqle	NM_009270
**-3.06**	**Sterol-C4-methyl oxidase-like**	**Sc4mol**	**AK005441**
**-2.98**	**3-hydroxy-3-methylglutaryl-Coenzyme A synthase 1**	**Hmgcs1**	**BI690696**
**-2.92**	**3-hydroxy-3-methylglutaryl-Coenzyme A synthase 1**	**Hmgcs1**	**BI690696**
**-2.92**	**3-hydroxy-3-methylglutaryl-Coenzyme A synthase 1**	**Hmgcs1**	**BI690696**
**-2.81**	**3-hydroxy-3-methylglutaryl-Coenzyme A synthase 1**	**Hmgcs1**	**BB705380**
**-2.74**	**Adenosine deaminase, RNA-specific, B2**	**Adarb2**	**BC004801**
**-2.67**	**Farnesyl diphosphate farnesyl transferase 1**	**Fdft1**	**BB028312**
**-2.45**	**Farnesyl diphosphate synthetase**	**Fdps**	**BI247584**
**-2.43**	**Cytochrome P450, family 51**	**Cyp51**	**NM_020010**
**-2.26**	**Farnesyl diphosphate farnesyl transferase 1**	**Fdft1**	**NM_010191**
**-1.71**	**7-dehydrocholesterol reductase**	**Dhcr7**	**NM_007856**
**-1.55**	**NAD(P) dependent steroid dehydrogenase-like**	**Nsdhl**	**BC019945**
**-1.45**	**Lanosterol synthase**	**Lss**	**C77434**
**-1.24**	**Cytochrome P450, family 51**	**Cyp51**	**BB004104**
**-1.07**	**Sterol-C5-desaturase (fungal ERG3, delta-5-desaturase) homolog (S. cerevisae)**	**Sc5d**	**AB016248**
-1.01	Transmembrane 7 superfamily member 2	Tm7sf2	BC014769
-0.98	Mevalonate kinase	Mvk	BC005606
**-0.93**	**StAR-related lipid transfer (START) domain containing 4**	**Stard4**	**AK014587**
-0.79	StAR-related lipid transfer (START) domain containing 4	Stard4	BB246854
**-0.77**	**Aldolase 3, C isoform**	**Aldoc**	**BC008184**
**-0.68**	**3-hydroxy-3-methylglutaryl-Coenzyme A reductase**	**Hmgcr**	**BB123978**
**-0.64**	**RIKEN cDNA 0610007P14 gene**	**0610007P14Rik**	**AK002308**
**-0.45**	**Insulin receptor substrate 4**	**Irs4**	**NM_010572**
-0.44	Proline-serine-threonine phosphatase-interacting protein 2	Pstpip2	AV229693
-0.34	Microtubule-associated protein 6	Mtap6	NM_010837
-0.33	Tachykinin receptor 3	Tacr3	NM_021382
-0.30	Sema domain, immunoglobulin domain (Ig), short basic domain,	Sema3b	BB116052

	secreted, (semaphorin) 3B		
-0.29	RIKEN cDNA 4833422M21 gene	4833422M21Rik	AV251026
**-0.29**	**Cyclin-dependent kinase inhibitor 1C (P57)**	**Cdkn1c**	**NM_009876**
-0.28	Sema domain, immunoglobulin domain (Ig), short basic domain, secreted, (semaphorin) 3A	Sema3a	NM_009152
**-0.27**	**Insulin-like growth factor binding protein 4**	**Igfbp4**	**NM_010517**
**-0.27**	**Selenoprotein K**	**Selk**	**AK007523**
-0.27	Paired box gene 5	Pax5	NM_008782
-0.25	Forkhead box K1	Foxk1	L26507
**-0.22**	**Epithelial membrane protein 2**	**Emp2**	**BE573195**
-0.21	Serine (or cysteine) peptidase inhibitor, clade A, member 3A	Serpina3a	NM_028740
**-0.21**	**Transforming growth factor alpha**	**Tgfa**	**M92420**
**-0.21**	**Capicua homolog (Drosophila)**	**Cic**	**AF363690**
-0.21	Apolipoprotein M	Apom	NM_018816
-0.21	Guanine nucleotide binding protein 13, gamma	Gng13	AB030194
**-0.19**	**Cysteine-rich secretory protein LCCL domain containing 1**	**Crispld1**	**AV299594**
**-0.17**	**Eyes absent 2 homolog (Drosophila)**	**Eya2**	**BC003755**
-0.17	Selectin, lymphocyte	Sell	M36005
-0.13	Mediator of RNA polymerase II transcription, subunit 25 homolog (yeast)	Med25	NM_029365
**-0.13**	**FYVE, RhoGEF and PH domain containing 4**	**Fgd4**	**AF402612**
0.12	Son cell proliferation protein	Son	BG067046
**0.14**	**Transcription factor 25 (basic helix-loop-helix)**	**Tcf25**	**BC025071**
0.16	Transmembrane protein 1	Tmem1	BM200437
**0.17**	**Calmodulin 1**	**Calm1**	**AU079514**
**0.18**	**Transmembrane protein 66**	**Tmem66**	**BC022616**
**0.19**	**F-box and WD-40 domain protein 2**	**Fbxw2**	**AK009893**
0.19	Aldo-keto reductase family 1, member A4 (aldehyde reductase)	Akr1a4	AI627032
**0.19**	**Xanthine dehydrogenase**	**Xdh**	**AV286265**
0.20	PX domain containing serine/threonine kinase	Pxk	BC016131
0.20	Annexin A7	Anxa7	NM_009674
0.21	Upstream binding protein 1	Ubp1	NM_013699
0.23	Bernardinelli-Seip congenital lipodystrophy 2 homolog (human)	Bscl2	AF069954
**0.24**	**Cyclin-dependent kinase 4**	**Cdk4**	**NM_009870**
0.25	Possible Acireductone dioxygenase 1		AU046270

**0.25**	**Possible Acireductone dioxygenase 1**		**AU046270**
0.27	Mesoderm development candiate 2	Mesdc2	NM_023403
**0.27**	**Tripartite motif protein 27**	**Trim27**	**NM_009054**
**0.29**	**ADP-ribosylation factor-like 6 interacting protein 1**	**Arl6ip1**	**AF223953**
0.29	Propionyl-Coenzyme A carboxylase, alpha polypeptide	Pcca	AY046947
0.30	ATP-binding cassette, sub-family B (MDR/TAP), member 6	Abcb6	NM_023732
0.30	IMP3, U3 small nucleolar ribonucleoprotein, homolog (yeast)	Imp3	NM_133976
0.32	SEC63-like (S. cerevisiae)	Sec63	C76103
0.33	Fibroblast growth factor receptor 4	Fgfr4	NM_008011
0.36	Topoisomerase (DNA) I	Top1	BG068053
0.37	Mannose-P-dolichol utilization defect 1	Mpdu1	NM_011900
**0.41**	**ADP-ribosylation factor-like 6 interacting protein 1**	**Arl6ip1**	**AF133669**
0.42	Phosphopantothenoylcysteine synthetase	Ppcs	NM_026494
**0.47**	**RIKEN cDNA 6430706D22 gene**	**6430706D22Rik**	**BQ173888**
0.48	RIKEN cDNA 4931406C07 gene	4931406C07Rik	AK016432
0.49	Folate receptor 2 (fetal)	Folr2	BC022108
0.51	Dehydrogenase/reductase (SDR family) member 8	Dhrs8	BB546344
**0.60**	**ATP-binding cassette, sub-family C (CFTR/MRP), member 3**	**Abcc3**	**AK006128**
0.70	Acetyl-Coenzyme A acyltransferase 1A	Acaa1a	NM_130864
**1.01**	**Aldehyde dehydrogenase family 3, subfamily A2**	**Aldh3a2**	**NM_007437**
