# Supplementary material for: The Sterolgene v0 cDNA microarray: a systemic approach to studies of cholesterol homeostasis and drug metabolism
Source: BMC Genomics. 2008 Feb 11;9:76. doi: 10.1186/1471-2164-9-76 (PMC2262072; doi:10.1186/1471-2164-9-76)
Supplement: Additional file 8 — Differentially expressed genes in mouse liver after phenobarbital treatment using the same probability of type I error as for Sterolgene array (Affymetrix GeneChip). Differentially expressed genes as detected by Affymetrix GeneChip using the same probability of type I error as for Sterolgene data (α = 0.1). Only genes that are also present in the Steroltalk array were considered in the analysis. Genes in bold are confirmed using RT-PCR, genes in italic coincide with the results from the Sterolgene platform. [file 1471-2164-9-76-S8.pdf]

**Log_2 ratio**	**Gene name**	**Gene symbol**	**GeneBank Acc. No.**
-0.58	ATP-binding cassette, g5	Abcg5	NM_031884
-0.33	scavenger receptor class B, member 1	Scarb1	NM_016741
-0.33	ATP-binding cassette, g8	Abcg8	AF324495
-0.28	nuclear receptor, 2f6	Nr2f6	NM_010150
**-0.21**	**SREBP cleavage activating protein**	**Scap**	**BI412871**
-0.18	apolipoprotein A-I	Apoa1	NM_009692
-0.15	vitamin D receptor	Nr1i1	AV290079
-0.14	sterol carrier protein 2, liver	Scp2	C76618
-0.14	ATP-binding cassette, b1b	Abcb1b	AI450248
-0.11	farnesyl diphosphate synthetase	Fdps	AV156959
-0.09	estrogen related receptor, alpha	Nr3b1	NM_007953
-0.05	ATP-binding cassette, b1a	Abcb1a	M30697
0.09	nuclear receptor, 1d2	Nr1d2	NM_011584
0.10	*nuclear receptor, 2c1*	*Nr2c1*	*NM_011629*
0.15	peroxisome proliferator activated receptor gamma	Pparg	NM_011146
0.16	membrane-bound transcription factor peptidase, site 1	Mbtps1	NM_019709
0.23	cytochrome P450, 2g1	Cyp2g1	NM_013809
0.54	ATP-binding cassette, c2	Abcc2	NM_013806
0.99	***aminolevulinic acid synthase 1***	***Alas1***	***BC022110***
1.14	*cytochrome P450, 2a4*	*Cyp2a4*	*NM_007812*
6.25	***cytochrome P450, 2b10***	***Cyp2b10***	***NM_009998***
