# Supplementary material for: The Sterolgene v0 cDNA microarray: a systemic approach to studies of cholesterol homeostasis and drug metabolism
Source: BMC Genomics. 2008 Feb 11;9:76. doi: 10.1186/1471-2164-9-76 (PMC2262072; doi:10.1186/1471-2164-9-76)
Supplement: Additional file 9 — Differentially expressed genes in mouse liver after cholesterol feeding using the same probability of type I error as for Sterolgene array (Affymetrix GeneChip). Differentially expressed genes as detected by Affymetrix GeneChip using the same probability of type I error as for Sterolgene data (α = 0.1). Only genes that are also present in the Steroltalk array were considered in the analysis. Genes in bold are confirmed using RT-PCR, genes in italic coincide with the results from the Sterolgene platform. [file 1471-2164-9-76-S9.pdf]

Log_2 ratio	Gene name	Gene symbol	GeneBank Acc. No.
-3.40	*squalene epoxidase*	*Sqle*	*NM_009270*
-3.12	sterol-C4-methyl oxidase-like	Sc4mol	AK005441
-2.60	farnesyl diphosphate synthetase	Fdps	BI247584
-2.30	***farnesyl diphosphate farnesyl transferase 1***	***Fdft1***	***NM_010191***
-1.77	7-dehydrocholesterol reductase	Dhcr7	NM_007856
-1.52	*phosphomevalonate kinase*	*Pmvk*	*BI713896*
-1.45	lanosterol synthase	Lss	C77434
-1.44	*NAD(P) dependent steroid dehydrogenase-like*	*Nsdhl*	*BC019945*
-1.31	mevalonate (diphospho) decarboxylase	Mvd	NM_138656
-1.21	*cytochrome P450, family 51*	*Cyp51a1*	*NM_020010*
-1.16	sterol-C5-desaturase (fungal ERG3, delta-5-desaturase) homolog (S. cerevisae)	Sc5d	AB016248
-1.04	*acyl-CoA synthetase short-chain family member 2*	*Acss2*	*NM_019811*
-0.99	mevalonate kinase	Mvk	BC005606
-0.66	acetyl-Coenzyme A acetyltransferase 2	Acat2	AV148646
-0.65	sterol regulatory element binding factor 2	Srebf2	BM123132
**-0.49**	**cytochrome P450, family 26, subfamily a, polypeptide 1**	**Cyp26a1**	**NM_007811**
-0.47	24-dehydrocholesterol reductase	Dhcr24	BC004738
-0.40	RAR-related orphan receptor alpha	Nr1f1	BI660199
-0.35	estrogen related receptor, beta	Esrrb	NM_011934
-0.34	cytochrome P450, family 17, subfamily a, polypeptide 1	Cyp17a1	NM_007809
-0.34	phenylalkylamine Ca2+ antagonist (emopamil) binding protein	Ebp	NM_007898
-0.23	steroidogenic acute regulatory protein	Star	L36062
-0.23	transcription factor 1	Tcf1	NM_009327
-0.22	vitamin D receptor	Vdr	AV290079
-0.18	SREBP cleavage activating protein	Scap	BI412871
0.07	cytochrome P450, family 2, subfamily a, polypeptide 12	Cyp2a12	NM_133657
0.15	ATP-binding cassette, sub-family B (MDR/TAP), member 1B	Abcb1b	NM_011075
0.16	retinoid X receptor alpha	Rxra	BQ175050
0.19	similar to glyceraldehyde-3-phosphate dehydrogenase	Gapd	M32599
0.19	ATP-binding cassette, sub-family B (MDR/TAP), member 7	Abcb7	BM119407
0.22	ATP-binding cassette, sub-family A (ABC1), member 1	Abca1	BB144704
0.22	membrane-bound transcription factor peptidase, site 1	Mbtps1	NM_019709
0.24	androgen receptor	Ar	NM_013476
0.25	cytochrome P450, family 2, subfamily d, polypeptide 22	Cyp2d22	BF683039
0.25	nuclear receptor subfamily 2, group F, member 2	Nr2f2	AI463873

0.26	ATP-binding cassette, sub-family B (MDR/TAP), member 4	Abcb4	NM_008830
0.46	ATP-binding cassette, sub-family C (CFTR/MRP), member 2	Abcc2	NM_013806
0.50	peroxisome proliferator activated receptor alpha	Ppara	BC016892
0.59	sterol regulatory element binding factor 1	Srebf1	AI326423
0.70	ATP-binding cassette, sub-family G (WHITE), member 8	Abcg8	AF324495
0.94	nuclear receptor subfamily 1, group D, member 2	Nr1d2	NM_011584
1.72	ATP-binding cassette, sub-family G (WHITE), member 5	Abcg5	NM_031884
