# Supplementary material for: The Sterolgene v0 cDNA microarray: a systemic approach to studies of cholesterol homeostasis and drug metabolism
Source: BMC Genomics. 2008 Feb 11;9:76. doi: 10.1186/1471-2164-9-76 (PMC2262072; doi:10.1186/1471-2164-9-76)
Supplement: Additional file 10 — Primer sequences used in quantitative RT-PCR analyses [file 1471-2164-9-76-S10.pdf]

Gene symbol	Primer/Probe sequence
Alas1	Forward: 5'-GGCCTCCCGGTCATCC-3'<br>Reverse: 5'-TGTTCTTAGCAGCATCGGCA-3'<br>Probe: 5'-FAM-CTGTCCGAGTCACATCATCCCTGTGC-TAMRA-3'
Fdft1[1]	Forward: 5'-CCAACTCAATGGGTCTGTTCCT-3'<br>Reverse: 5'-TGGCTTAGCAAAGTCTTCCAACT-3'
Cyp2b10	Forward: 5'-CAATGTTTAGTGGAGGAACTGCG-3'<br>Reverse: 5'-CACTGGAAGAGGAACGTGGG-3'<br>Probe: 5'-FAM-CCCAGGGAGCCCCCCTGGA-TAMRA-3'
Cyp3a11	Forward: 5'- AGAACTTCTCCTTCCAGCCTTGTA-3'<br>Reverse: 5'- GAGGGAGACTCATGCTCCAGTTA-3'<br>Probe: 5'-FAM-CTAAAGGTTGTGCCACGGGATGCAGT-TAMRA-3'
Cyp8b1[3]	Forward: 5'- AAGGCTGGCTTCCTGAGCTT-3'<br>Reverse: 5'- AACAGCTCATCGGCCTCATC-3'
Cyp51a1	Forward: 5'-ACGCTGCCTGGCTATTGC-3'<br>Reverse: 5'- TTGATCTCTCGATGGGCTCTATC-3'
Sc4mol	Assay-on-demand, Applied Biosystems
Srebp2[1]	Forward: 5'- GCGTTCTGGAGACCATGGA-3'<br>Reverse: 5'- ACAAAGTTGCTCTGAAAACAAATCA-3'
ApoA1[4]	Forward: 5'- TCCTCCTTGGGCCAACA-3'<br>Reverse: 5'- GAACCCAGAGTGTCCCAGTTT-3'
ApoA1[8]	Forward: 5'- CCCAGTCCCAATGGGACA-3'<br>Reverse: 5'- CAGGAGATTCAGGTTCAGCTGTT-3'
Actb	Forward: 5'- CCGTGAAAAGATGACCCAGATC-3'<br>Reverse: 5'- CACAGCCTGGATGGCTACGT-3'
Scap[1]	Forward: 5'- ATTTGCTCACCGTGGAGATGTT-3'<br>Reverse: 5'- GAAGTCATCCAGGCCACTACTAATG-3'
Scarb1[5]	Forward: 5'- TCAGAAGCTGTTCTTGGTCTGAAC-3'<br>Reverse: 5'- GTTCATGGGGATCCCAGTGA-3'
Ppara[6]	Forward: 5'- CCTCTTCCCAAAGCTCCTTCA-3'<br>Reverse: 5'- CGTCGGACTCGGTCTTCTTG-3'
Cyp26a1[7]	Forward: 5'- CGGTTCAGCTTCATTCCATT-3'<br>Reverse: 5'- CAGTGGGGCTTGTCTTCATT-3'
Cyp7a1[5]	Forward: 5'- CAGGGAGATGCTCTGTGTTCA-3'<br>Reverse: 5'- AGGCATACATCCCTTCCGTGA-3'
Cyp1a2[4]	Forward: 5'- TGGAGCTGGCTTTGACACAG-3'<br>Reverse: 5' – CGTTAGGCCATGTCACAAGTAGC-3'
Car[9]	Forward: 5'- CAGGGTTCCAGTACGAGTTTTG-3'<br>Reverse: 5'- AGGCTCCTGGAGATGCAGTC-3'

FAM: 6-carboxyfluorescein; TAMRA: 6-carboxy-tetramethyl-rhodamine;
[1]Yang, J., Goldstein, J.L., Hammer, R.E., Moon, Y.A., Brown, M.S. and Horton, J.D. (2001) Decreased lipid synthesis in livers of mice with disrupted Site-1 protease gene. *Proc Natl Acad Sci U S A*, **98**, 13607-13612; [2]Waalkes, M.P., Liu, J., Chen, H., Xie, Y., Achanzar, W.E., Zhou, Y.S., Cheng, M.L. and Diwan, B.A. (2004) Estrogen signaling in livers of male mice with hepatocellular carcinoma induced by exposure to arsenic in utero. *J Natl Cancer Inst*, **96**, 466-474; [3]Kok, T., Hulzebos, C.V., Wolters, H., Havinga, R., Agellon, L.B., Stellaard, F., Shan, B., Schwarz, M. and Kuipers, F. (2003) Enterohepatic circulation of bile salts in farnesoid X receptor-deficient mice: efficient intestinal bile salt absorption in the absence of ileal bile acid-binding protein. *J Biol Chem*, **278**, 41930-41937; [4] Fon Tacer, K., Kuzman, D., Seliskar, M., Pompon, D., Rozman, D. (2007) TNF-alpha interferes with lipid

homeostasis and activates acute and proatherogenic processes. *Physiol Genomics*, 31(2), 216-27; [5]Plösch, T., Kok, T., Bloks, V.W., Smit M.J., Havinga, R., Chimini, G., Groen, A.K., Kuipers, F. (2002) Increased hepatobiliary and fecal cholesterol excretion upon activation of the liver X receptor is independent of ABCA1. *J Biol Chem*, 277 (37), 33870-7; [6]Patel, D.D., Knight, B.L., Wiggins, D., Humpreys, S.M., Gibbons, G.F. (2001) Disturbances in the normal regulation of SREBP-sensitive genes in PPARα-deficient mice. *J Lipid Res*, 42: 328-337; [7]Kimura, N., Tamura, T.A., Murakami, M. (2005) Evaluation of the performance of two carbodiimide-based cyanine dyes for detecting changes in mRNA expression with DNA microarrays. Biotechniques, 38(5), 797-806; [8]RTPrimerDB database; [9]Maglich, J.M., Stoltz, C., M., Goodwin, B., Hawkins-Brown, D., Moore, J.T., Kliewer, S.A. (2002) Nuclear pregnane x receptor and constitutive androstane receptor regulate overlapping but distinct sets of genes involved in xenobiotic detoxification. *Mol Pharmacol*, 62(3): 638-46.
